# Ontogenic Expression Pattern and Genetic Polymorphisms of the Fatty Acid Transport Protein 4 (*FATP4*) Gene in Chinese Chicken Populations

**DOI:** 10.3390/ijms13066820

**Published:** 2012-06-05

**Authors:** Yan Wang, Qing Zhu, Ling Yang, Yi-Ping Liu

**Affiliations:** College of Animal Science and Technology, Sichuan Agricultural University, Ya’an, Sichuan 625014, China; E-Mails: wangyan519723614@yahoo.com.cn (Y.W.); zhuqing5959@163.com (Q.Z.); www.yangling999@sina.com (L.Y.)

**Keywords:** *FATP4* gene, single nucleotide polymorphism, mRNA expression, carcass trait

## Abstract

In the current research, the polymorphism of *FATP4* gene was analyzed in Erlang Mountainous chickens. A total of nine genetic variants were identified by *FATP4* gene sequencing analysis across the chicken samples. Significant associations (*p* < 0.05) were observed for two SNPs (g.5608778C>T and g.5608814G>A in exon 6) with certain carcass traits (such as live weight, carcass weight, eviscerated weight) in S01 and S05 populations, respectively. Meanwhile, in S05 population, haplotype 3 (T-G) and haplotype 2 (C-A) were associated with higher and lower partial carcass traits such as live weight, carcass weight, eviscerated weight and semi-eviscerated weight, respectively. Moreover, we investigated the expression profile of this gene during ontogenesis in Mountainous black-boned chicken. Quantitative real-time PCR analysis showed that *FATP4* mRNA had the highest expression level in small intestine tissue over all other tissues examined. The *FATP4* mRNA levels presented remarkable developmental changes with age in the various tissues. These results suggested that the *FATP4* gene might play an important role in controlling chicken carcass traits.

## 1. Introduction

As the key metabolites for energy generation and storage, long-chain fatty acids (LCFAs) are crucial for cell function and activity. Cellular uptake and metabolism of long-chain fatty acids (LCFAs) are believed to be regulated by a variety of membrane-associated proteins ring [1] and are related to body composition. Previous studies have identified several candidate proteins that play active roles in this process, including fatty acid translocase (FAT/CD36) [2], fatty acid binding protein (FABPs) [3,4], long chain fatty acyl-CoA synthetases (ACSL) and fatty acid transport protein (FATPs) [5]. Specifically, FATPs proteins have been shown to participate in the transport of exogenous fatty acids into the cell. At present, six *FATP* isoforms have been identified. *FATP1* is the major type in adipose tissue and is also present in heart and skeletal muscle [6–8]; *FATP2* is mainly expressed in the liver and kidneys [1,6]; *FATP3* is found in the liver, pancreas, and lungs [9,10]; *FATP5* expression is prevalent in the liver [1,6]; and *FATP6* is specifically expressed in the heart [11,12]. Interestingly, *FATP4* is the only FATP found in the small intestine, but it is also expressed in adipose, brain, liver, skin and heart tissues [13]. In addition, *FATP4* is involved in the absorption of dietary lipids [14] and may serve as a potential anti-obesity target [15].

The *FATP4* gene (the other name is *SLC27A4*), which was first cloned and characterized in mice by Herrmann *et al.* [16], contains an open reading frame consisting of 1929 bp and encodes 643 amino acid residues. The exon-intron structure of murine *FATP4* resembles that of human *FATP4*, indicating this gene is highly conserved [16]. Disruption of *FATP4* in mice leads to neonatal mortality [15], and *FATP4* heterozygous mice show decreased fatty acid uptake into enterocytes *ex vivo*, but no effect on fat absorption *in vivo*, possibly due to the large excess capacity for fat absorption in the small intestine. Lobo *et al.* [17] make use stable *FAT4* knockdown 3T3-L1 cell lines to investigate the role of *FATP4* in facilitating adipocyte fatty acid metabolism, they found that *FATP4* exhibited reduced triacylglycerol deposition and increased basal lipolysis.

*FATP4* polymorphisms and their association with metabolic alterations and dyslipidemia have been studied in humans as well. *FATP4* was considered to be associated with ichthyosis prematurity syndrome [18], insulin resistance [19] and related disorders such as obesity, and it is negatively correlated with growth [20]. Variations in the *FATP4* gene could affect body mass index and other obesity-related traits to different extents. In human, a mutation in exon 3, causing an amino acid exchange (p.G209S) in the *FATP4* protein, was associated with lower body mass index (BMI), triacylglycerol and insulin concentrations, systolic blood pressure and homeostasis model assessment index (HOMA) [19]. Meanwhile, *FATP4* expression had also been linked to markers of insulin resistance and obesity in humans [21]. In mice, Gimeno *et al.* [12] found that heterozygous *FATP4* deletion mutants shown decreased fatty acid uptake into enterocytes *ex vivo*, but do not have decreased fat absorption *in vivo*. Similarly, Bower *et al.* [22] also found that increasing *FATP4* mRNA and protein levels in adipocytes of obese subjects correlates with a higher LCFA uptake rates in African-American and Caucasian women.

Most previous studies concerning the tissue distribution of *FATP4* have primarily been performed on human and mouse tissues. However, many important issues regarding *FATP4* expression remain unresolved in chicken. First, whether the expression pattern is the same between mammals and birds is still not known. Second, the ontogenesis of chicken *FATP4* mRNA expression has not been defined. This is vital to transporters because differences in expression during early development and adult maturation have been shown to result in differential susceptibility to injury. Third, there is currently no information associating *FATP4* gene polymorphisms with chicken carcass traits. Thus the purpose of the current study was to determine (1) the expression levels of *FATP4* mRNA in various chicken tissues, (2) the ontogenic expression pattern of the chicken *FATP4* gene and (3) polymorphisms in *FATP4* and their association with chicken carcass traits.

## 2. Results

### 2.1. Expression of *FATP4* among Chicken Tissues

Quantitative PCR analysis showed that *FATP4* mRNA was expressed in all eight MB chicken tissues. At 84 days, the mRNA levels of the chicken *FATP4* gene were extremely high in the small intestine, followed by the leg muscle weight (LMW), brain, abdominal fat weight (AW), breast muscle weight (BMW), liver, subcutaneous fat (SFT) and heart (Table 1). The relative expression level of *FATP4* in small intestine at 84 days was significantly higher (*p* < 0.01) than other all tissues and no significant expression differences among other tissues.

### 2.2. Ontogenic Expression of *FATP4* in Chicken

**Figure 1:**
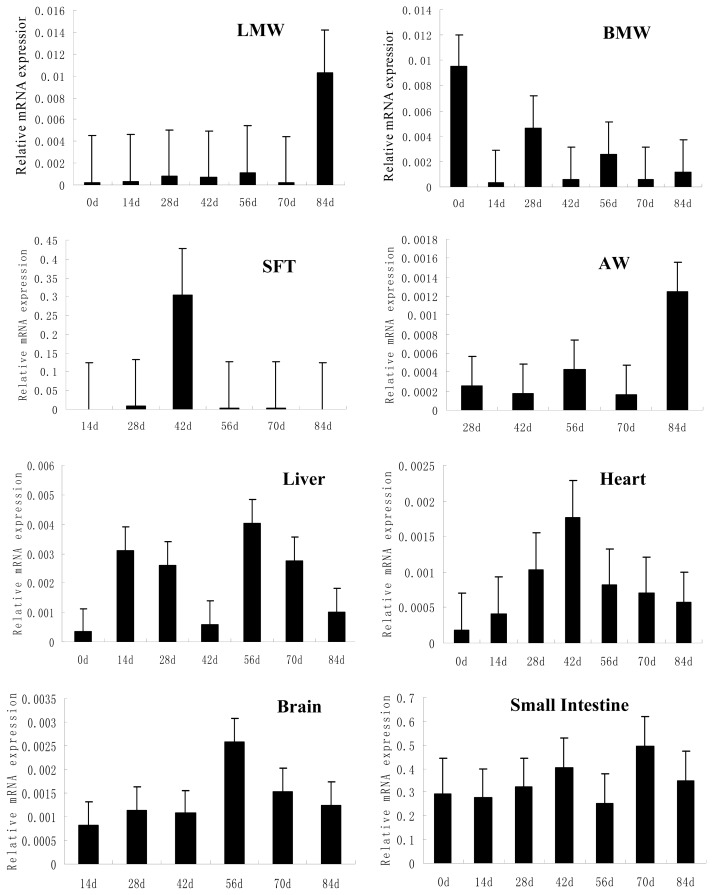
Relative *FATP4* mRNA expression levels of different Mountainous Black-boned chicken tissues at different ages. Data were normalized with β-actin in each sample and are presented as means ± SD. LMW = leg muscle; BMW = breast muscle; SFT = subcutaneous fat; AW = abdominal fat.

To clarify whether the *FATP4* mRNA expression had an age-related expression pattern, we investigated the ontogenic expression of this gene in eight MB chicken tissues. As shown in Figure 1, *FATP4* mRNA expression levels showed no difference in leg muscle and subcutaneous fat at various days of age (*p* > 0.05). The *FATP4* mRNA in breast muscle, contrastingly, had the highest expression on 0 day and the lowest expression on 14 days (*p* < 0.01). In abdominal fat, the expression level of *FATP4* mRNA on 84 days was significantly higher than the expression on 28 days, 42 days and 70 days by 79.3%, 85.8% and 86.9%, respectively. In liver tissue, the level of *FATP4* mRNA showed no difference between 0 day and each of the three growth points on 28 days, 42 days and 84 days. However, the expression of *FATP4* mRNA on 56 days was significantly higher than the expression level on 0 day by 91.7% (*p* < 0.01) (Figure 1). In heart tissue, the *FATP4* mRNA levels presented a unimodal distribution pattern with increasing age peaked on 42 days, but there were no significant differences at various ages (*p* > 0.05). In brain tissue, the *FATP4* mRNA expression exhibited a “rise-decline-rise-decline” developmental change, and its expression on 56 days was significantly higher than at other times (*p* < 0.01). Finally, in small intestine, *FATP4* was expressed at 56 days with a low level then increased to a peak at 70 days and down at 84 days. In addition, although the expression level of *FATP4* mRNA on 70 days was higher than the expression on 56 days and 84 days by 48.9% and 29.8%, respectively, there were no differences at various days of age (*p* > 0.05).

### 2.3. Identification of Genetic Variants in Chicken *FATP4* Gene

The direct sequencing of these fragments in representative samples showing different bands on SSCP gels (see Materials and Methods section) yielded a total of nine genetic variants. We named these polymorphisms relative to their respective positions on sequences of chicken genomic chromosome 17 (reverse strand; GenBank accession number NC_006104.2, GI: 118135643), and we deposited the SNPs in GenBank under dbSNP numbers ss345102396-ss345102405. The locations of the nine SNPs are as follows: one in intron 7 (g.5608272C>G, synonymous), one in intron 6 (g.5608695 G>A, synonymous), one in exon 9 (g.5607545T>C, synonymous), one in intron 10 (g.5606740C>T, synonymous), one in exon 13 (g.5606005A>G, synonymous), two in exon 14 (g.5605454C>T, synonymous; g.5605481G>A, synonymous), and the remaining two (which are also the only ones causing amino acid changes) in exon 6 (g.5608814G>A, p.A443T; g.5608778C>T, p.R455*).

We further estimated the likelihood that the two missense variants could cause a putatively functional impact on the *FATP4* protein by using the cSNP analysis tool in PANTHER. The subPSEC score was −2.18124, and the *p*_deleterious_ value was 0.30603 for variant p.A443T. According to these results, the functional effect of variant p.A443T is unlikely to be harmful, but it may somewhat change normal gene functions. Variant p.R455* was found to be a nonsense mutation by analysis with PANTHER, so it likely alters normal gene function. Therefore, to confirm our hypothesis, we focused on these two variants with putatively functional roles in S01 and S05 chicken populations.

### 2.4. Association Analysis

Table 2 presents the association results of g.5608778C>T (p.R455*) and g.5608814G>A (p.A443T) genotypes with chicken carcass traits in two different populations. With the exception of g.5608778C>T in S01 populations, the allele frequencies of both g.5608778C>T and g.5608814G>A were not deviated from Hardy-Weinberg equilibrium (HWE) in two populations. In the S01 population, the g.5608778C>T SNP was significantly associated with BMW and with LMW (*p* < 0.05). All other traits were not significantly associated with this SNP (*p* > 0.05). In the S05 population, g.5608778C>T was significantly associated with all carcass traits except for AW. Genotype CC was associated with lower values of BW (*p* = 0.0003), CW (*p* = 0.0004), SEW (*p* = 0.0015), EW (*p* = 0.0006), zBMW (*p* = 0.0156) and LMW (*p* = 0.0047). Similarly, genotypes of g.5608814G>A in exon 6 were also associated with some chicken carcass traits in different populations (Table 2). In the S01 population, chickens with the GG genotype had the highest BW (*p* = 0.0229), CW (*p* = 0.0147) and EW (*p* = 0.0272), while the other traits were not significantly associated with this SNP. In the S05 population, the g.5608814G>A SNP was significantly associated with BW, CW, SEW and EW (*p* < 0.05), whereas it was negatively associated (*p* > 0.05) with BMW, LMW and AW. In addition, we found that the two SNPs were significantly associated with BW, CW, EW and SEW when both S01 and S05 were included in the analysis (data not shown).

To further define the haplotype structures of the *FATP4* gene, haplotype blocks were analyzed using the Haploview program. Two most common LD measures, D′ and, were used to determine the extent of LD in the *FATP4* gene. The D′ values for combinations of these two SNPs were 0.45 and *r*^2^ values were 0.12, which indicated that these two polymorphisms were linked.

Therefore, to discern the potential effect of haplotypes on carcass traits, the g.5608778C>T and g.5608814G>A haplotypes were analyzed in the S05 population. Four possible haplotypes were discerned: haplotype 1 = C-G with a frequency of 31.3%, haplotype 2 = C-A with a frequency of 24.6%, haplotype 3 = T-G with a frequency of 26.8%, and haplotype 4 = T-A with a frequency of 17.3%. In the S01 population, only haplotype 1 was well represented (data not shown) with the other haplotype frequencies equaling less than 10%; therefore, this population was not fully informative for a haplotype association analysis. Table 3 shows the estimated regression coefficient of the haplotype substitution effect for the carcass traits. For the S05 population, haplotype 3 (T-G) was the most favorable haplotype due to its association with higher BW, CW, EW, SEW, BMW and LMW (*p* < 0.05). On the contrary, animals with haplotype 2 (C-A) showed lower BW, CW, EW and SEW (*p* < 0.05). Haplotype 1 (C-G) showed lower BMW and LMW (*p* = 0.0421 and *p* = 0.0461, respectively), but had no correlation with other carcass traits. Haplotype 4 (T-A) did not correlate with any carcass traits (*p* > 0.05), possibly due to the small number of chickens with this haplotype.

## 3. Discussion

FATPs protein with six isoforms, one of which is *FATP4*, it plays an important role in fatty acid utilization. Understanding the tissue distribution of *FATP4* may help identify its impact on organ-specific fatty-acid transportation. Previous studies concerning the tissue distribution of *FATP4* were limited to a few tissues and focused on human and mice. Therefore, to our knowledge, this study represents the first attempt to characterize the mRNA expression profile of *FATP4* in chickens. This study has generated important information regarding mRNA levels in different chicken tissues as well as differences between mammalian and avian genetics.

FATP4 mRNA was expressed in all the tissues examined. Consistent with previously published reports, FATP4 mRNA expression is highest in the small intestine, and is also present in brain, liver, heart, skeletal muscle and adipose tissues [6,23–25]. *FATP4* expression in the brain suggests that FATPs may be responsible for the activation of long-chain polyunsaturated fatty acid that is important for brain function [26]. Moreover, the strong expression of chicken *FATP4* in the small intestine suggests that it may also be required for efficient uptake of fatty acids by these cells and confirm the *FATP4* gene was seen in bird species like other species. Meanwhile, it is interesting to note that the expression level of the *FATP4* mRNA in chicken breast muscle was higher than that of leg muscle at 0 day, 14 days, 28 days, 56 days and 70 days (Figure 1).

In order to obtain a comprehensive understanding of the characteristics of *FATP4*, we examined the ontogeny of *FATP4* in specific tissues of chicken during development. Notable findings in our study include: (1) *FATP4* was expressed in all examined tissues and throughout chicken development; (2) The expression level of *FATP4* changed in different ways in the tissues as the chickens aged. We observed that the expression level of *FATP4* mRNA in subcutaneous fat peaked at 42 days and then sharply decreased by subsequent time points. Contrastingly in mice, Feng demonstrated that the mRNA expression of *FATP4* in fatty liver increased at 2 weeks, and was especially high at 12 weeks after a high-fat diet [27]. Schmuth *et al.* (2005) also reported *FATP4* to be highly expressed in subcutaneous fat and sebaceous glands [28].

As this study is the first describing the expression of *FATP4* in chicken tissues over the course of development, these findings broaden our understanding of the mechanisms underlying normal fatty acid transportation in different tissues, opening the door for future research on *FATP4* as a potential target in preventing or reversing obesity.

Our current study shows that the *FATP4* gene is associated with some chicken carcass traits. We found that in the S01 population, the g.5608778C>T SNP was significantly associated with BMW and LMW, but not with other traits. However, in the S05 population, g.5608778C>T was significantly associated with all carcass traits except for AW. Similarly, genotypes of g.5608814G>A were significantly associated with BW, CW and EW in the S01 populations and significantly associated with all carcass traits except for AW in the S05 population.

It might be contested that the results of SNP association conflict with each other for S01 and S05 in regards to several traits such as body type, body weight and speed of growth. Two explanations are possible. The first is that the two populations have different backgrounds and characteristics. The S01 chickens grow faster than S05, while all other examined carcass trait parameters were lower than for S05. Conversely, S05 is a larger, meatier chicken and, thus, has been subjected to intensive artificial selection for its favorable commercial traits, such as carcass weight, eviscerated weight, and breast muscle weight. The second hypothesis proposes that additional polymorphisms possibly affecting the analyzed traits ought to be considered in the two populations.

The traditional approach for studying a candidate gene with complex traits generally uses one or a few SNPs; however, haplotype analysis is thought to be more powerful than single SNP analysis in searching for genetic determinants of complex diseases or traits [29]. However, the effectiveness of haplotype phasing compared to single SNP analysis has been debated for several years. Daly *et al.* demonstrated that the haplotype or haplotype block provided a practical solution to resolve some problems such as unsatisfied and obscured localization information [30]. Conversely, Clark concluded that if the causal connection between SNP and a the phenotype is truly driven by just a single SNP, the haplotype approach may perform worse than the one SNP analysis [31]. Therefore, to dissect the effects of the haplotypes on carcass traits, we analyzed the g.5608778C>T and g.5608814G>A haplotypes in the S05 population. Four possible haplotypes were discerned. Haplotype 3 (T-G) was most favorable with respect to BW, CW, EW, SEW, BMW and LMW (*p* < 0.05). In most of the situations tested, haplotype 2 (C-A) showed lower BW, CW, EW and SEW. Values for haplotype 4 (T-A) were not associated to any carcass traits. So, the current data shows that associations of haplotypes with carcass traits were more accurate than those of single SNP. This result implies that different SNPs interact with each other and that haplotypes generally provide more information than individual SNPs [32].

In summary, our results show that the two SNPs (g.5608778C>T and g.5608814G>A) and haplotypes were associated with certain carcass traits (BW, CW, EW and SEW) in the studied chicken populations. Therefore, these markers have practical implications: (1) these SNPs could be used for marker-assisted selection in both populations and (2) the *FATP4* gene could be directly or indirectly involved in affecting carcass trait phenotypes. However, to avoid any possible bias due to the relatively small population size in this study, a future investigation with a larger sample size and more breeds is necessary.

## 4. Experimental Section

### 4.1. Animal

The Mountainous Black-boned chicken (MB) is a well-known bird with spotty feathers, black or yellow skin and favorable meat quality natively bred in Sichuan Province, China. MB chickens were used in the expression study, and all chickens were raised on an experimental farm for poultry breeding at the Sichuan Agricultural University (Ya’an, China) under the same conditions. For the ontogeny studies, different tissues including heart, liver, brain, leg muscle, breast muscle, abdominal fat, subcutaneous fat and the small intestine from male chicken were collected at 0 day, 14 days, 28 days, 42 days, 56 days, 70 days and 84 days of age (five samples of each age). Fresh tissues were identified, excised, then immediately frozen in liquid nitrogen and stored at −80 °C until mRNA isolation took place. For quantification of the overall tissue expression profile of chicken *FATP4* mRNA, five adult male chicken at growth point 84 days were used, and the above eight tissues were collected and frozen in the same way.

Two groups of chicken (S01 and S05) were selected for association analysis between DNA markers and carcass traits. S01 and S05 belong to Erlang Mountainous chickens. The first group was made up by 128 S01 chickens (65 females and 63 males) with yellow partridge plumage, blue shanks and white skin. These chickens had a favorable quality of meat and grew at a fast rate. The second group of chickens was made of 136 S05 chickens (69 females and 67 males) with big bodily forms and a high quantity of meat. Eggs of the two breeds were hatched in different incubators under the same conditions on 15 March 2008; the chickens were kept in single-breed pens and were slaughtered on 15 June 2008. During the growth period, all birds had free access to food and water ad libitum under the same temperature and lighting conditions. From birth to 3 weeks of age, chickens were fed, ad libitum, a starting diet containing 2.90 megacalories (Mcal) of metabolizable energy (ME)/kg of body weight and 20.5 g of crude protein (CP)/kg of body weight. Birds were fed a grower diet (3.00 Mcal of ME/kg and 18.5 g of CP/kg) from 4 to 6 weeks of age and were transferred to growing pens at 7 weeks. Before the chickens were slaughtered, a blood sample was collected and stored at −20 °C for each one.

### 4.2. Measurement of Carcass Traits

The two groups of chicken used for the association study were measured for performance-tested traits at the Sichuan Dahen Poultry Breeding Company. At the age of 90 days, live weight (BW) was measured on chickens restricted from feed for 12 h. After slaughter on that same day, the carcass traits were measured, including carcass weight (CW), eviscerated weight (EW), semi-eviscerated weight (SEW), breast muscle weight (BMW), leg muscle weight (LMW) and abdominal fat weight (AW). CW was measured of chilled carcasses removed of feathers. SEW was measured of carcasses removed of the trachea, esophagus, gastrointestinal tract, spleen, pancreas, and gonad. EW was measured of the semi-eviscerated carcasses after additional removal of the head, claws, heart, liver, gizzard, glandular stomach and abdominal fat.

### 4.3. RNA Extraction, cDNA Synthesis and Quantification of *FATP4* mRNA Expression

Total RNA was extracted from chicken tissues using the TRIzol reagent (Invitrogen) and was treated with RNase-free DNase I using the RNase-free DNase Set (TakaRa Biotechnology Co. Ltd., Dalian, China). For each sample, about 1 μg of total RNA was reverse transcribed using the ImProm-II Reverse Transcription System (TakaRa Biotechnology Co. Ltd., Dalian, China). The reaction was performed in a volume of 10 μL containing 5× PrimerScript Buffer, 10 mM of each dNTPs, 40 U/μL RNase Inhibitor and 2.5 μM oligo-dT Primer. The reverse transcription was maintained at 30 °C for 10 min, and then at 45 °C for 25 min. The cDNA product was stored at −20 °C.

The *FATP4* mRNA expression levels in different tissues collected at different developmental stages of male MB chicken were quantified by the SYBR Green I assay on an IQ5 real-time PCR thermal cycle instrument (Bio-Rad, USA). Chicken mRNA sequences of the β-actin gene (GenBank accession number NM_205518) and SLC27A4 (accession number XM_415504) were retrieved from GenBank. Primers were designed using Oligo 6.0 (Table 1). Reactions were performed in triplicate in a volume of 12.5 μL containing 10 mM Tris-HCl (pH 8.3), 50 mM KCL, 3.5 mM MgCL_2_, 50 U/mL Takara ExTaq™ R-PCR (Takara, Biotechnology Co. Ltd., Dalian, China), 0.2 μM of each specific primer and 0.25 μL/300 diluted SYBR Green I (Takara, Biotechnology Co. Ltd., Dalian, China). The cycling condition consisted of an initial denaturation cycle for 5 min at 95 °C, then 35 cycles of 30 s at 95 °C, 40 s at 61 °C, and 45 s at 72 °C, ending with 8 min at 72 °C. Gene expression levels were quantified relatively to the expression of β-actin using the comparative 2^−ΔΔCT^ method [33]. Then, data of the mRNA expressions were subjected to ANOVA and/or *t*-test to determine the difference between tissues/growth points using SAS software 6.12 (SAS Institute Inc.). The *p*-value for significance was set at *p* ≤ 0.01.

### 4.4. Identification of FATP4 Genetic Variants

Genomic DNA was extracted from whole blood using the standard phenol/chloroform method. According to the UCSC Genome Browser information for the chicken genome, May 2006 Assembly [34] that was retrieved by searching for the *Gallus gallus FATP4* sequence (GenBank accession number XM_415504), the chicken *FATP4* gene contains 15 exons. Fifteen pairs of primers were designed to amplify these exons (and partially cover some introns) (Table 4). Primer synthesis was completed by Shanghai Yingjun Biotechnology Co. Ltd. (Shanghai, China). PCR reactions were performed using the Gene Amp PCR System 9700 (Bio-Rad, USA) thermal cycler in a final volume of 10 μL reaction containing 10–100 ng genomic DNA, 10 pmol of each primer and 5 μL 2× Master mix (including Mg^2+^, dNTPs, *Taq* DNA polymerase; Beijing TIAN WEI Biology Technique Corporation, Beijing, China). The amplification condition included an initial step of denaturation for 5 min at 94 °C; 35 cycles of 45 s at 94 °C, 35 s at 55 °C (or other apt annealing temperature as shown in Table 1), and 45 s at 72 °C; then ended with a final extension step for 7 min at 72 °C. Genetic variants in the *FATP4* genomic sequence were analyzed by using the PCR-SSCP (single strand conformation polymorphism) method. Briefly, after a denaturation at 99 °C for 10 min, 3 μL of PCR product was rapidly cooled on ice and then loaded on 12% acrylamide/bisacrylamide (39:1) gels. Electrophoresis was performed at 120–150 V for 13–15 h in 1× TBE buffer, and gels were silver-stained. Three DNA samples showing different patterns on SSCP gel were further amplified and purified, and then sequenced by Shanghai Yingjun Biology Technique Corporation (Shanghai, China).

### 4.5. Data Analysis

We performed *in silico* functional predictions for *FATP4* gene nonsynonymous variations using PANTHER (Protein analysis through evolutionary relationships) [35], which estimates the likelihood of a particular amino acid substitution to cause a functional impact on a protein. It calculates the substitution position-specific evolutionary conservation (subPSEC) score and the probability of a deleterious effect on protein function caused by a given variant (*p*_deleterious_). PANTHER subPSEC scores are continuous values from 0 (neutral) to about −10 (most likely to be deleterious), and a subPSEC score of −3 corresponds to a *p*_deleterious_ of 0.5 [36].

Association analyses were carried out independently for the S01 and S05 chickens. The general linear model (GLM) procedure of SAS 6.12 (Statistical Analysis Systems Institute Inc., Cary, NC) was used to test associations between the genotyped markers and carcass traits. The model is as follows:

$${\text{Y}}_{\text{ijk}}=\mu +{\text{S}}_{\text{i}}+{\text{G}}_{\text{j}}+{\text{Sire}}_{\text{k}}+{\text{e}}_{\text{ijk}}$$

where Y_ijk_ is the trait measured in animal k, S_i_ is the effect of the ith sex category, G_j_ is the effect of the genotype j for SNP in the *FATP4* gene, Sire_k_ is the effect of the sire k and e_ijk_ is the residual random effect associated with animal k.

In addition, the identified SNPs (g.5608778C>T and g.5608814G>A) in this gene were tested for Hardy-Weinberg equilibrium and the linkage disequilibrium (LD) between SNPs was tested by the Haploview [37]. Two parameters, including D′ and *r*^2^, were used to characterize LD patterns within the *FATP4* gene. The value of D′ > 0.33 and *r*^2^ > 0.1 were applied as the criterion for meaningful LD [36]. Haplotypes between the g.5608778C>T and g.5608814G>A were constructed using the PHASE program v. 2.0 [38]. Haplotypes were analyzed using the model applied for the single marker association test with consideration for animals having 0, 1, or 2 copies of the haplotype in question. The PROC REG procedure of SAS (version 8.02, SAS Institute Inc.) was used to perform the analysis. Significant associations were declared when *p* < 0.05.

## 5. Conclusion

In summary, we characterized the *FATP4* gene expression in eight tissues of MB chicken and performed association analyses to discern the effect of *FATP4* gene variants on chicken carcass traits. Data presented in our study provide a basis for future studies on chicken *FATP4* function and will potentially lead to a better understanding of the regulatory mechanisms involved in chicken small intestine and skeletal muscles. The *FATP4* gene variants (g.5608778C>T and g.5608814G>A) that had a significant association with carcass traits may be used as genetic markers linking to quantitative trait loci or to a major gene with effects on carcass traits. Further analyses of the effects of *FATP4* SNPs are essential to confirm the association between the alleles or haplotypes and chicken carcass traits in other chicken breeds and lines.

## Figures and Tables

**Table 1:** Expression of *FATP4* mRNA at 84 days among chicken tissues.

Tissue	LMW	BMW	SFT	AW	Liver	Brain	Heart	Small intestine
Expression level	0.0101 ± 0.0163 ^b^	0.0011 ± 0.0163 ^b^	0.0009 ± 0.0163 ^b^	0.0012 ± 0.0163 ^b^	0.0010 ± 0.0163 ^b^	0.0014 ± 0.0163 ^b^	0.0005 ± 0.0163 ^b^	0.3494 ± 0.0167 ^a^

LMW = leg muscle; BMW = breast muscle; SFT = subcutaneous fat; AW = abdominal fat.

**Table 2:** Associations between the *FATP4* g.5608778C>T and g.5608814G>A genotypes and carcass traits in S01 and S05 chicken populations.

Breed/Line	Traits 1	g.5608778C>T genotype			*p* value	g.5608814G>A genotype			*p* value
	
		CC (*n* = 28)	CT (*n* = 82)	TT (*n* = 18)		GG (*n* = 36 )	GA (*n* = 71)	AA (*n* = 21)	
**S01**	BW (g)	1807.85 (86.64)	1925.18 (50.94)	1720.00(108.06)	0.1684	1925.83 (75.19)	1779.57 (53.92)	2077.14 (98.45)	0.0229
	CW (g)	1608.75 (80.21)	1710.25 (47.75)	1497.65(102.94)	0.1397	1718.06 (69.43)	1564.70 (50.89)	1853.81 (90.91)	0.0147
	SEW (g)	1508.93 (133.01)	1597.16 (79.19)	1827.06(170.70)	0.3329	1611.11 (118.06)	1573.66 (86.54)	1716.67 (154.58)	0.7223
	EW (g)	1252.14 (65.13)	1330.51 (38.78)	1160.53 (83.60)	0.1747	1342.36 (56.57)	1218.81 (41.47)	1430.95 (74.07)	0.0272
	BMW (g)	132.57 (7.06)	104.59 (4.20)	103.25 (9.34)	0.0028	104.86 (6.50)	111.77 (4.80)	117.86 (8.51)	0.4597
	LMW (g)	183.75 (11.54)	150.57 (6.87)	140.19 (15.26)	0.0268	148.00 (10.39)	156.44 (7.68)	172.86 (13.61)	0.3513
	AW (g)	46.75 (5.32)	47.27 (3.17)	42.75 (7.05)	0.8426	50.17 (4.68)	43.94 (3.46)	48.62 (6.13)	0.5287
	*p* (HWE)	0.001				0.1578			

	**Traits**	**CC (*n*** **= 43)**	**CT (*n*** **= 66)**	**TT (*n*** **= 27)**	***p*** **value**	**GG (*n*** **= 45)**	**GA (*n*** **= 68)**	**AA (*n*** **= 23)**	***p*** **value**

**S05**	BW (g)	1867.91 (65.66)	2035.15 (53.00)	2308.89 (82.86)	0.0003	2171.56 (65.80)	1917.35 (53.53)	2125.22 (92.04)	0.0077
	CW (g)	1677.32 (60.84)	1819.62 (49.11)	2074.44(76.78)	0.0004	1950.22 (60.86)	1717.21 (49.51)	1900.00 (85.12)	0.0090
	SEW (g)	1574.53 (61.69)	1691.97 (49.79)	1940.74 (77.85)	0.0015	1826.67 (61.55)	1609.04 (50.07)	1746.09 (86.10)	0.0225
	EW (g)	1307.21 (49.86)	1428.41 (40.25)	1625.07 (62.92)	0.0006	1532.11 (49.66)	1338.49 (40.39)	1495.65 (69.46)	0.0072
	BMW (g)	104.42 (3.90)	113.26 (3.15)	122.67 (4.93)	0.0156	116.56 (3.85)	107.07 (3.13)	119.61 (5.39)	0.0575
	LMW (g)	137.40 (6.78)	156.06 (5.48)	173.22 (8.56)	0.0047	163.71 (6.75)	144.07 (5.49)	161.78 (9.45)	0.0532
	AW (g)	60.74 (10.04)	59.74 (8.11)	80.67 (12.67)	0.3514	68.82 (9.87)	63.36 (8.03)	57.70 (13.82)	0.7984
	*p* (HWE)	0.8539				0.7540			

The values for each trait refer to the least square means (LSM), with their standard errors displayed in parenthesis;

1 BW = live weight (g); CW = carcass weight (g); SEW = semi-eviscerated weight (g); EW = eviscerated weight (g); BMW = breast muscle weight (g); LMW = leg muscle weight (g); AW = abdominal fat weight (g); *p* (HWE) = The values of Hardy-Weinberg test.

**Table 3:** Estimated regression coefficients and standard errors (SE) of the haplotype substitution effect on carcass traits in the S05 chicken population.

	Haplotype 1 (C-G) 2		Haplotype 2 (C-A) 2		Haplotype 3 (T-G) 2		Haplotype 4 (T-A) 2	
	
Traits 1	Regression Coefficient (SE)	*p* value	Regression Coefficient (SE)	*p* value	Regression Coefficient (SE)	*p* value	Regression Coefficient (SE)	*p* value
BW (g)	−115.324 (61.308)	0.0621	−196.131 (67.671)	0.0044	185.277 (59.189)	0.0021	140.408 (81.034)	0.0854
CW (g)	−99.764 (56.724)	0.0809	−180.269 (62.528)	0.0046	167.779 (54.752)	0.0026	122.698 (74.942)	0.1039
SEW (g)	−84.273 (57.175)	0.1428	−173.478 (62.974)	0.0067	164.280 (55.099)	0.0034	92.618 (75.611)	0.2228
EW (g)	−82.293 (46.354)	0.0781	−142.542 (51.211)	0.0062	136.279 (44.773)	0.0028	96.495 (61.301)	0.1178
BMW (g)	−7.240 (3.528)	0.0421	−5.351 (3.998)	0.1830	7.466 (3.478)	0.0336	6.649 (4.692)	0.1588
LMW (g)	−12.463 (6.190)	0.0461	−12.954 (6.968)	0.0652	16.979 (6.027)	0.0056	9.238 (8.250)	0.2648
AW (g)	−0.818 (9.006)	0.9277	−12.846 (10.054)	0.2035	7.253 (8.868)	0.4149	6.194 (11.868)	0.6026

1 BW=live weight (g); CW = carcass weight (g); SEW=semi-eviscerated weight (g); EW= eviscerated weight (g); BMW = breast muscle weight (g); LMW= leg muscle weight (g); AW = abdominal fat weight (g);

2 Haplotypes are indicated with the respective allele status of SNPs g.5608778C>T and g.5608814G>A.

**Table 4:** Primer pairs used in this study.

Primer name	Primer sequence (5′→3′)	Annealing temperature (°C)	Product length (bp)	Amplified region
**Primer pairs for measuring chicken** ***FATP4*** **gene expression**				
FATP4-F	CATCACCATCTCCAACTCCAAG	61	126	1188–1313
FATP4-R	GACTCAGGGCTTCCTTCTCCT			
β-actin-F	GAGAAATTGTGCGTGACATCA	60	180	685–836
β-actin-R	CCTGAACCTCTCATTGCCA			
**Primer pairs for screening chicken** ***FATP4*** **gene polymorphisms**				
P-1F	TCCGGGATCCCACGAGAC	54	243	5614016–5614358
P-1R	ACGGCATTGGTGGCATAGCA			
P-2F	ACGAGGCGGTTATTC	55	309	5613698–5614006
P-2R	GTCCCACCAGAGTCGCATTT			
P-3F	CGCCGCGCTAGAAGT	57	239	5613326–5613664
P-3R	CCCGCTGGGAGCTGTAGT			
P-4F	CAGGCCAAGATGCTGCGTCTGGCT	55	215	5610456–5610670
P-4R	ACACACCCCAGCGCACAGTT			
P-5F	GTCCTGCTGCGGGTGAAGTG	55	302	5609605–5609906
P-5R	GAATTCACCAGGGCCGTCT			
P-6F	CCGGTGCTCTTTCTCCATCT	55	313	5608527–5608939
P-6R	CTCTGCTGCTGAAGTCTGCC			
P-7F	CGCTGCATGTGTGACCTTGT	55	233	5608184–5608418
P-7R	GCCATGCGGAAATACCTG			
P-8F	GGCCCTGCTTCTGACAT	55	152	5608087–5608238
P-8R	GTCCCAAGGGCACACGTTAC			
P-9F	GGGAACTCGGGGTACTGA	55	245	5607424–5607668
P-9R	GACAGACAGGCAGAACGAGT			
P-10F	TTGCCCCTGCTAGATTGT	55	204	5606940–5607143
P-10R	AGGCTGCAGTTGCACTCGGT			
P-11F	CATGGCGTGCGTTAAGAT	55	166	5606652–5606817
P-11R	AAGCCAATGGGGTACACT			
P-12F	CCTTGGGCATGAGCGGTC AC	55	141	5606311–5606451
P-12R	TTGCTGGTGGCTGACTGATT			
P-13F	GCTCCTCTCACACCTCGTT	55	232	5605920–5606151
P-13R	CCCTCCCCTCTCAGTTAC			
P-14F	AGGGTGTCGCTGGTAAAC	55	308	5605358–5605665
P-14R	GTGCAGGAACCGTAGGA			
P-15F	GAAGATGGAGCTGCGTAA	55	247	5604728–5604974
P-15R	CTAGTGTGCCTTTATACC			

The forward and reverse primers are marked by “F” and “R” in the primer names, respectively; The locations of the *FATP4* gene exons in the genomic region were scored according to the UCSC Chicken Genome Browser May 2006 Assembly [34], using the *Gallus gallus FATP4* gene sequence (GenBank accession number XM_415504).
